# Dosimetric comparison of HyperArc and InCise MLC‐based CyberKnife plans in treating single and multiple brain metastases

**DOI:** 10.1002/acm2.14404

**Published:** 2024-05-27

**Authors:** Liying Zhu, Shengnan Dong, Lei Sun, Yixuan Xiao, Yihua Zhong, Mingyuan Pan, Yang Wang

**Affiliations:** ^1^ Radiation Oncology Center Huashan Hospital Fudan University Shanghai China; ^2^ Radiation Oncology Center Henan Province Hospital of TCM Zhengzhou China; ^3^ Department of Neurosurgery CyberKnife Center Huashan Hospital Fudan University Shanghai China

**Keywords:** brain metastases, CyberKnife, HyperArc, multi‐leaf collimator

## Abstract

**Background and purpose:**

This study aimed to compare the dosimetric attributes of two multi‐leaf collimator based techniques, HyperArc and Incise CyberKnife, in the treatment of brain metastases.

**Material and methods:**

17 cases of brain metastases were selected including 6 patients of single lesion and 11 patients of multiple lesions. Treatment plans of HyperArc and CyberKnife were designed in Eclipse 15.5 and Precision 1.0, respectively, and transferred to Velocity 3.2 for comparison.

**Results:**

HyperArc plans provided superior Conformity Index (0.91 ± 0.06 vs. 0.77 ± 0.07, *p* < 0.01) with reduced dose distribution in organs at risk (*D*_max_, *p* < 0.05) and lower normal tissue exposure (V4Gy–V20Gy, *p* < 0.05) in contrast to CyberKnife plans, although the Gradient Indexes were similar. CyberKnife plans showed higher Homogeneity Index (1.54 ± 0.17 vs. 1.39 ± 0.09, *p* < 0.05) and increased *D*_2%_ and *D*_50%_ in the target (*p* < 0.05). Additionally, HyperArc plans had significantly fewer Monitor Units (MUs) and beam‐on time (*p* < 0.01).

**Conclusion:**

HyperArc plans demonstrated superior performance compared with MLC‐based CyberKnife plans in terms of conformity and the sparing of critical organs and normal tissues, although no significant difference in GI outcomes was noted. Conversely, CyberKnife plans achieved a higher target dose and HI. The study suggests that HyperArc is more efficient and particularly suitable for treating larger lesions in brain metastases.

## 1 INTRODUCTION

Brain metastases are the most prevalent intracranial malignancies in adults, with approximately 20%−40% of patients with solid tumors developing brain metastases during their illness.^1^, ^2^ Stereotactic radiosurgery (SRS) is crucial in the treatment of brain metastases, offering local control rates comparable with those of surgical resection. For patients with single brain metastases, SRS has been shown to improve survival and control of locally treated metastasis compared with whole‐brain radiation therapy (WBRT).^3^

The concept of SRS was introduced by neurosurgeon Leksell, employing the Gamma Knife (GK) equipped with Cone collimators and Co^60^ radioactive sources to produce Gamma rays, while patients were immobilized using an invasive stereotactic frame.^4^

With advancements in linear accelerators, more efficient external beam radiation for SRS became possible. Subsequently, several technologies were developed, including the x‐ray Knife and CyberKnife (CK, Accuray Inc, Madison, USA).^5^ The x‐ray knife, equipped with small‐size circular collimators on a C‐arm Linac, delivers high‐energy beams in coplanar or non‐coplanar arrangements.^6^ Meanwhile, CK combines a lightweight Linac with a robotic arm for brain metastases irradiation, utilizing real‐time imaging for motion tracking during treatment.^7^, ^8^ The robotic arm can automatically correct deviations, precisely positioning the circular collimated beam with six degrees of freedom.

The use of frameless double mask immobilization devices, surface guided radiotherapy (SGRT), and image guided radiotherapy (IGRT) enables correction of positional errors to less than 1 mm.^9^ Additionally, micro multi‐leaf collimators (mMLC) enhance target conformity compared with circular collimators, allowing for the precise delivery of doses to larger and more complex targets while sparing normal tissue (NT) and reducing treatment time.^10^, ^11^

Consequently, the InCise™ MLC, featuring submillimeter accuracy in beam delivery on the CK platform, was developed. Studies by Jang and Murai have demonstrated that intracranial SRS using the InCise™ MLC is dosimetrically feasible and reduces treatment time by 30%−40%.^12^, ^13^ McGuinness reported improved homogeneity and higher conformity in targets with MLC‐based plans,^14^ while another study found that Iris and InCise^™^ produced equivalent SBRT plans in terms of dosimetry, radiobiology, and organs at risk (OARs) sparing.^15^

HyperArc (HA), a SRS technique based on the TrueBeam platform (Varian, USA), automatically sets the isocentric position, non‐coplanar field arcs, and collimator angles for brain metastases. The ‟Dry‐Run’’ function aid in avoiding potential collisions. HA provides significantly higher conformity and rapid dose fall‐off compared with conventional volumetric modulated arc therapy (VMAT) plans.^16^, ^17^ Recent studies have shown that HA achieves superior dosimetric quality in various intracranial and extracranial plans, including boosting SRT for Glioblastoma, hippocampal sparing whole‐brain RT, and SRS for C‐spine metastases.^18^, ^19^, ^20^

Previous studies comparing HA and CK, as well as other techniques, for single and multiple brain metastases revealed that HA plans significantly reduce treatment time and are comparable with CK plans in sparing normal brain tissue, especially in larger and more complex cases.^21^, ^22^, ^23^, ^24^ However, these studies optimized CK plans with circular collimators. Given the differences in target shaping between cones and MLC, a comparison between two MLC‐based techniques might be more appropriate.

Therefore, this study selected 17 patients with single or multiple brain metastases. HA and InCise MLC‐based CK were used for treatment plans. The parameters of dose distribution for target volume and OAR were compared and analyzed to explore the clinical application value of MLC‐based techniques in treating brain metastases.

## 2 MATERIALS AND METHODS

### 2.1 Patient selection

A retrospective analysis was conducted on 17 patients (6 men and 11 women) who underwent CK treatment for brain metastases at our center. The study received approval from the institutional review board (IRB), and all patients provided informed consent prior to radiotherapy. The age range of the patients was 46−76 years; their clinical characteristics are summarized in Table 1. Tumor volumes varied from 2.2 to 35.5 cc, with a median volume of 10.75 cc.

**TABLE 1:** Clinical characteristics of patients.

Number	Gender	Age (years)	Volume of planning target volume, cc	Number of lesions
1	M	76	15	1
2	F	53	3.3	1
3	F	59	11.3	1
4	F	64	5	1
5	M	72	2.4	1
6	M	59	5.4	1
7	F	62	19.8	2
8	F	58	5.4	2
9	M	70	12.8	2
10	F	63	3.7	3
11	F	46	6.6	3
12	F	60	8.8	3
13	F	69	35.5	3
14	M	55	2.2	3
15	M	71	10.8	4
16	F	47	28.7	4
17	F	53	6.1	5

Patients were immobilized using a customized thermoplastic mask, and thereafter, simulation based on computed tomography (CT, Toshiba 64 Slice, Japan) was conducted. The imaging range extended from the top of the skull to the lower edge of the T1 vertebra, with a slice thickness of 1 mm. Subsequently, a magnetic resonance imaging (MRI) scan was performed using a GE 750 W system (USA) with a slice thickness of 2 mm. The CT scans employed iohexol contrast (Omnipaque, Amersham, UK), and the MRI scans utilized dimeglumine gadopentetate contrast (dimeglumine gadopentetate injection, Beijing, China). The CT and MRI images were subsequently co‐registered in the treatment planning system (TPS).

Furthermore, to assess the maximum quality potential of each system for a more equitable comparison, we designed plans using several concentric spheres with diameters of 10, 15, 20, 25, and 30 mm in a Head and Neck phantom (Accuracy, USA) specifically developed for SRS end‐to‐end (E2E) testing. This phantom, available at our center for scanning, includes a polymethyl methacrylate (PMMA) ball with a diameter of 32 mm inside the skull.^25^

### 2.2 Treatment plans for CK

**FIGURE 1:**
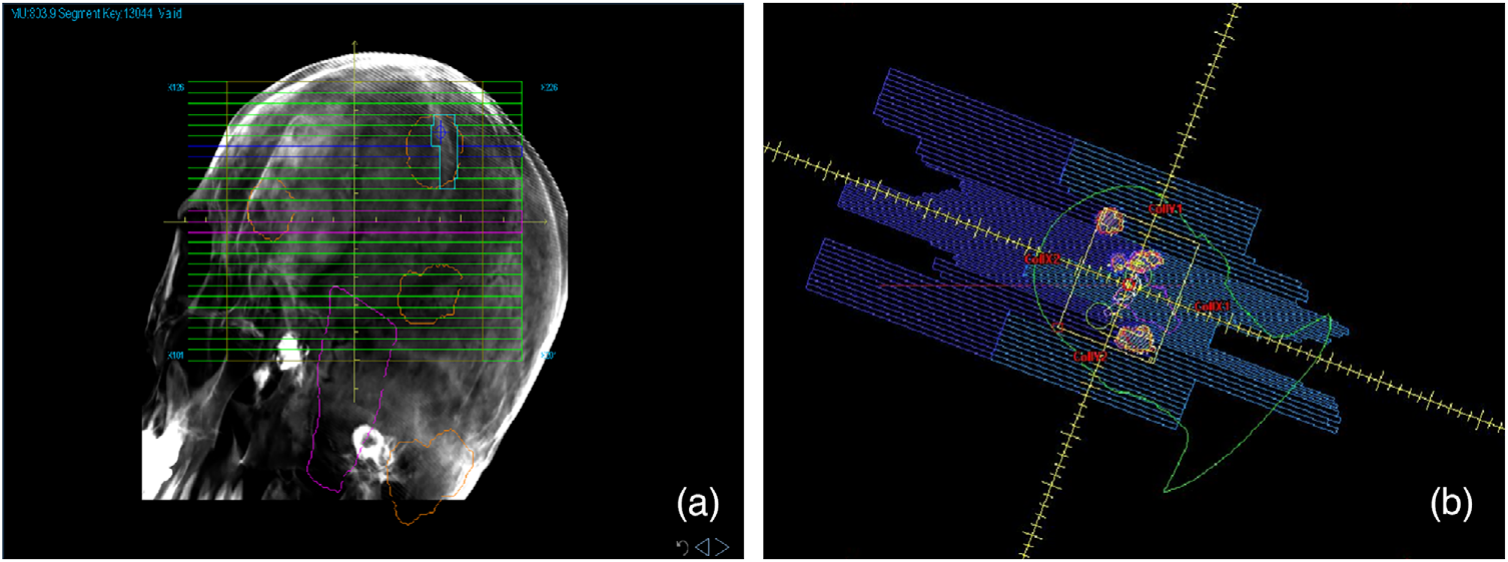
Visualization of one of segment shape and structures in CK plans (a) and HA plans (b).

The gross target volume (GTV) was delineated by clinicians based on enhanced MRI and was expanded by 1 mm to create the planning target volume (PTV). Treatment plans were designed using the Precision software (Version 1.0, Accuray Inc, Sunnyvale, USA) with the M6 CK system equipped with an Incise MLC and a 6 MV linear accelerator. The leaves of the Incise MLC measure 3.85 mm at an 800 mm Source Skin Distance (SSD) with a positioning error of less than 0.05 mm. The maximum clinical field size is 115 mm × 100 mm, and the minimum field size is 7.6 mm × 7 mm, as depicted in Figure 1a. A Ray‐Tracing algorithm was utilized, and the calculation resolution was set to high.

The GTV was delineated based on enhanced MRI, and the PTV was created with a 1 mm external expansion. The prescribed dose was 24 Gy/3fx, requiring the prescription dose to cover at least 95% of the PTV with the prescription isodose line set between 60% and 70% in the CK plan. In the premise of maintaining the clinical quality of the plans, segments, beams, and nodes were minimized to reduce treatment time to the extent possible. The dose distribution for the PTV and the dose limits to OARs conformed to our center's requirements, which include a maximum dose (*D*_max_) of less than 18 Gy to the brainstem and less than 17 Gy to the optic pathways.

In the Precision system, the PMMA sphere within the phantom was auto‐detected in Bulb‐Cube mode and isotropically shrunk to diameters ranging from 30 to 10 mm. E2E plans for each sphere were developed by several physicists to strictly avoid operator bias, adhering to Accuracy's instructions (the prescription isodose line of 420 cGy was set at 70%.^26^ No other compromises or constraints were applied except for auto shells. During optimization, the dosimetric quality demanded a coverage of more than 99.5%, a RTOG CI between 0.9 and 1.1, and a GI of less than 3, which remained consistent during priority adjustments in the Eclipse system.

### 2.3 Treatment plans for HA

**FIGURE 2:**
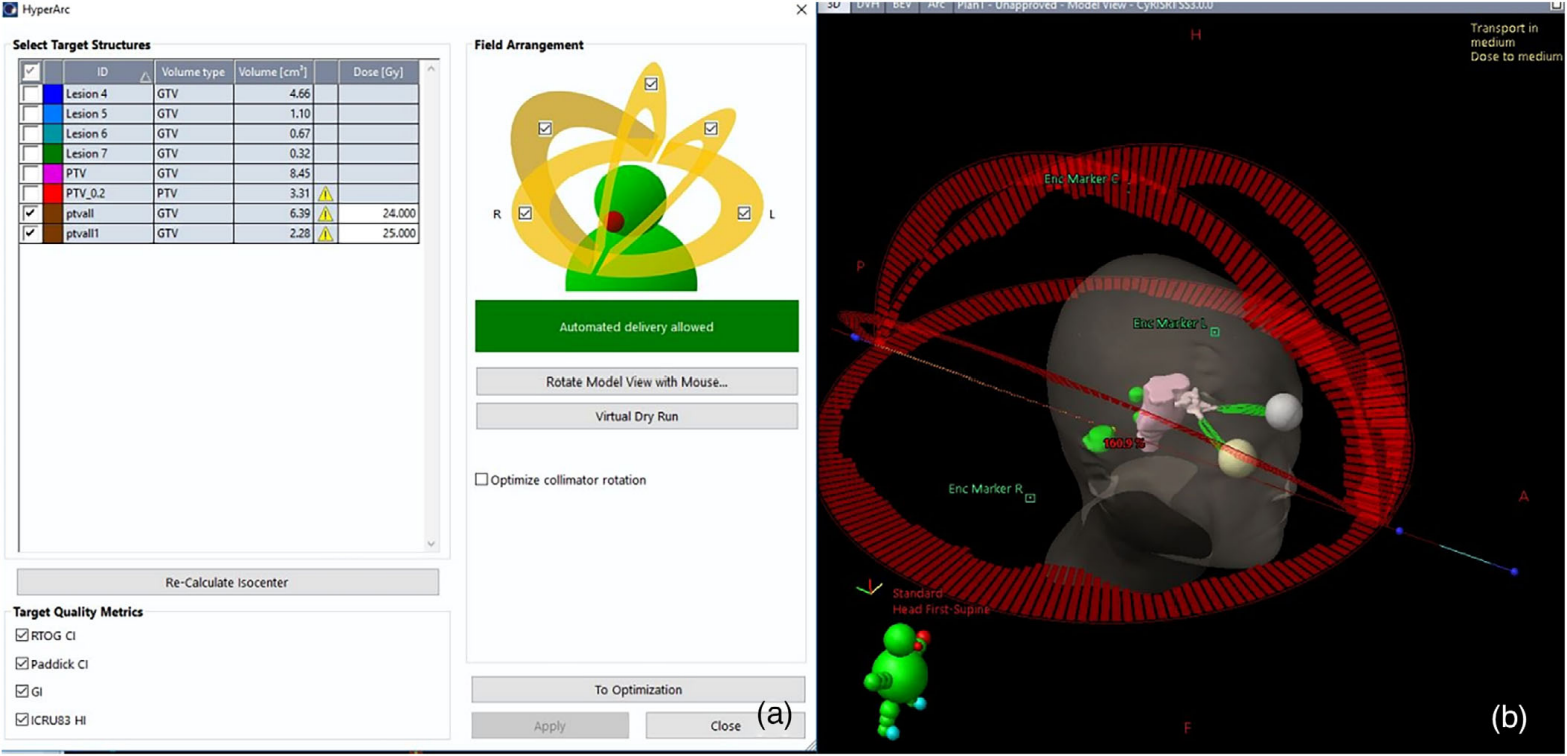
A screenshot for the HA plans in Eclipse (a). With dry run, HyperArc can perform collision check to select partial arcs (b).

All cases were subsequently re‐planned using Eclipse version 15.6 (Varian Medical Systems, Palo Alto) based on the TrueBeam STx linear accelerator, which features a 2.5‐mm leaf‐width multi‐leaf collimator (MLC), as shown in Figure 1b. The dose rate was set at 1400 MU/min for a 6 MV photon beam. HyperArc technology can automatically set the isocenter, arc fields, collimator angles, and couch angle based on the selected target volumes. A total of four arcs are available for HyperArc, including three non‐coplanar arcs and one coplanar arc, all sharing a single isocenter. The configuration was one full coplanar arc with a couch rotation of 0°, and three non‐coplanar arcs with couch rotations of 315°, 45°, and 90°, as shown in Figure 2a. The AcurosXB algorithm (Varian, V.15.6.06) was employed for plan optimization, with the dose grid size set at 1.25 mm. A normal tissue objective for SRS‐NTO was automatically applied to all HA plans. The Dry Run function was utilized to visually demonstrate the movement of the gantry and the couch, thereby preventing potential collisions during patient treatment, as shown in Figure 2b. The target prescription of 24 Gy/3Fx, a coverage of 95%, and the OAR dose limit remained consistent with the CK plans. Additionally, the maximum dose to the planning target volume (PTV) did not exceed 150% of the prescribed dose.

### 2.4 Plan design and evaluation

Both HA and CK plans were exported in DICOM format and imported into Velocity version 3.2 (Varian Medical Systems, Palo Alto, CA) for comparative analysis.

The parameters compared for the target volume in HA and CK plans include the homogeneity index (HI), Paddick conformity index (pCI), and gradient index (GI).

The calculation formulas are as follows: $HI=\frac{D_{\max}}{D_{\text{prescribed}}}$, $pCI=\left(\frac{V_{\text{t,ref}}}{V_{t}}\right)\times \left(\frac{V_{\text{t,ref}}}{V_{\text{ref}}}\right)$, $GI=\frac{PV_{50\%}}{PV}$, where *V*_t_ represents the volume of the target, *V*_t,ref_ denotes the volume of the target covered by the prescription isodose, and *V*_ref_ signifies the volume covered by the prescription isodose.^27^ PV_50%_ indicates the volume covered by the 50% prescription isodose.^28^ *D*_max_ and *D*_prescribed_ represent the maximum dose and the prescription dose, respectively. The doses at *D*_2%_ and *D*_98_%, which identify hot and cold spots in the target, along with *D*_50%_, have also been compared.

For sparing OARs, the *D*_max_ for each critical organ and the volume ranges from *V*_2Gy_ to *V*_20Gy_ in normal tissue (the volume of brain excluding the PTV) were assessed. Additionally, monitor units (MUs) and treatment time were evaluated to compare treatment efficiency.

### 2.5 Statistical methods

The statistical analysis of all data was conducted using SPSS version 22.0 (IBM, Chicago, IL, USA). Given that the data did not conform to a normal distribution, a Wilcoxon matched pairs signed rank test was employed for the analysis. The statistical data were expressed as mean ± standard deviation. A *p*‐value of less than 0.05 was considered statistically significant.

## 3 RESULTS

**FIGURE 3:**
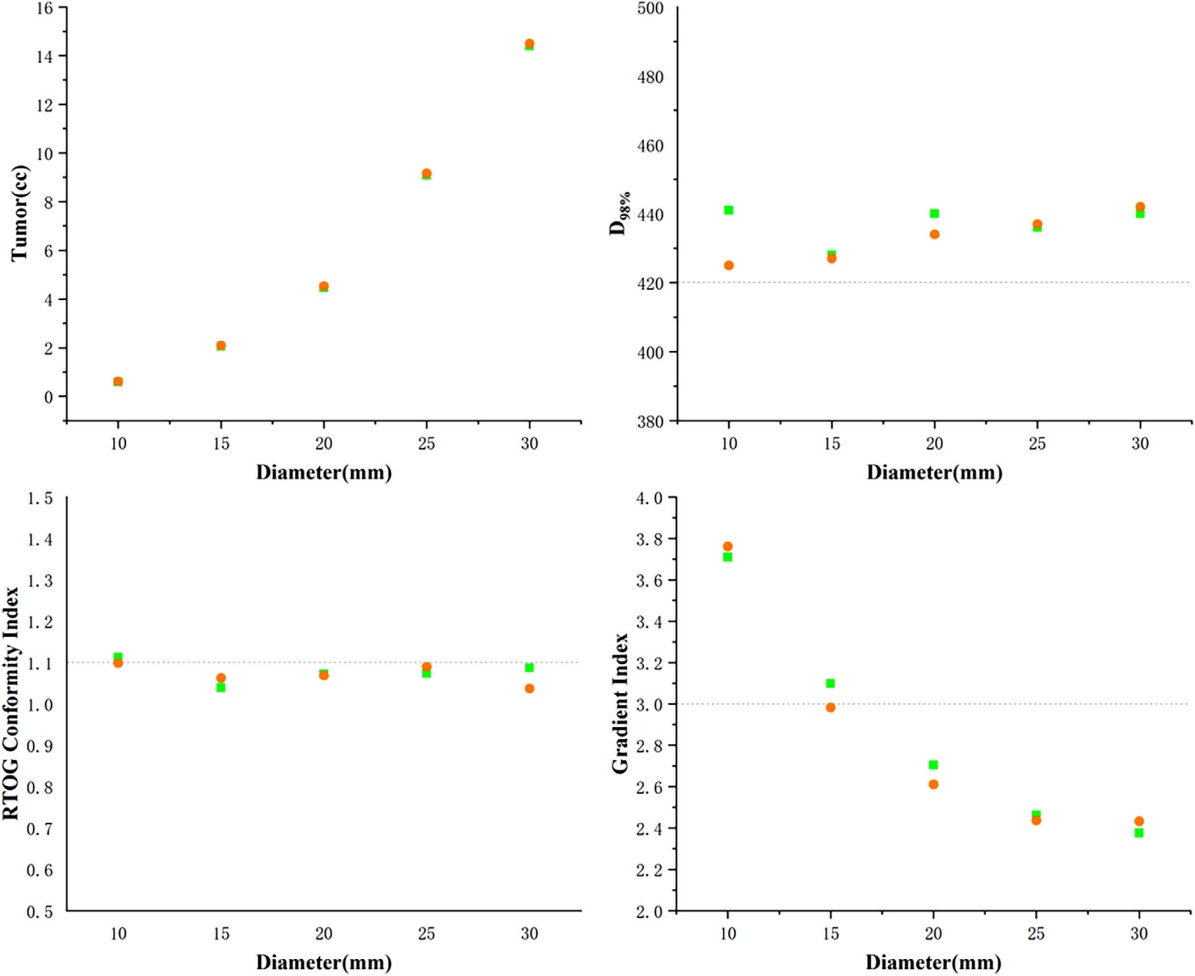
Scatter diagrams of HA and CK performance of phantom plans in Tumor volume, *D*_98%_, RTOG CI, and GI, with X axis represents sphere diameters. Green spots represent the HA plans and orange spots represent the CK plans.

The dosimetric performance of the two systems in single sphere phantom plans is depicted in Figure 3. All plans in Eclipse were normalized to achieve the same coverage of 420 cGy as obtained by Precision. Our findings indicated that with variations in the calculation algorithm and grid size, a tumor volume deviation ranging from 0.6% to 3% was noted as the volume decreased.^29^ Regarding the quality index, the mean deviations of D98%, RTOG CI (PIV/TV), and GI were 0.9%, 0.7%, and 0.9% respectively, with no statistically significant differences observed (*p* > 0.05). The results suggest that both HA and CK can produce clinically acceptable plans for targets ranging from 15 to 1 cc in the absence of adjacent lesions or OARs, although the quality deteriorated with decreasing volume.

**FIGURE 4:**
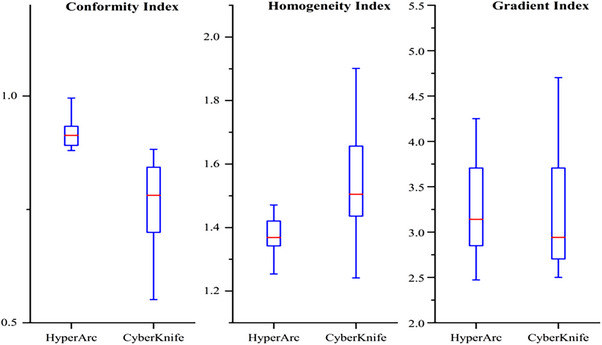
Boxplots of dosimetric parameters of CI, HI, and GI for tumor volume of HA and CK plans.

The dosimetric parameters of HA and CK plans are summarized in Table 2. Figure 4 presents the results of the dosimetric comparisons for the HI, Conformity Index (CI), and GI. Compared with the CK plans, the HA plans offered a lower HI (1.39 ± 0.09 for HA vs. 1.54 ± 0.17 for CK) and a higher CI (0.91 ± 0.06 for HA vs. 0.77 ± 0.07 for CK), and lower MUs (4619.29 ± 2949.67 for HA vs. 15 524.60 ± 8607.41 for CK). However, in terms of GI (3.34 ± 0.72 for HA vs. 3.27 ± 0.77 for CK), the difference was not statistically significant.

**TABLE 2:** Dosimetric parameters for CyberKnife and HyperArc plans of 17 patients.

	Dosimetric parameters	All (*n* = 17)				
		HyperArc plans		CyberKnife plans		*p‐*value
		Mean	Std	Mean	Std	
Tumor	HI	1.39	0.09	1.54	0.17	0.025*
	CI	0.91	0.06	0.77	0.07	0.000*
	GI	3.34	0.72	3.27	0.77	0.501
	*D*_2%_(Gy)	33.01	1.93	36.09	3.46	0.028*
	*D*_98%_(Gy)	23.19	0.41	23.74	1.97	0.492
	*D*_50%_(Gy)	28.20	0.79	30.27	3.37	0.049*
	Beaming on time(min)	4.03	2.58	36.41	11.59	0.000*
	MUs	4619.29	2949.67	15 524.60	8607.41	0.004*
OAR	*D*_max_Eye_	2.03	1.98	3.47	3.55	0.309
	*D*_max_Optic nerve_	2.68	1.92	4.22	3.24	0.011*
	*D*_max_Opticchiasm_	2.71	1.82	3.78	2.96	0.035*
	*D*_max_Brainstem_	6.59	6.75	8.32	6.94	0.004*
NT	*V*_2Gy_	576.78	461.83	649.89	358.68	0.062
	*V*_4Gy_	176.73	145.85	304.26	242.54	0.000*
	*V*_6Gy_	90.55	68.36	156.89	152.14	0.000*
	*V*_8Gy_	58.50	42.93	90.56	89.11	0.000*
	*V*_10Gy_	42.38	30.50	58.15	52.51	0.000*
	*V*_12Gy_	32.93	23.55	41.56	33.15	0.001*
	*V*_14Gy_	26.68	19.12	32.08	23.27	0.001*
	*V*_16Gy_	22.15	16.06	26.22	18.78	0.002*
	*V*_18Gy_	18.60	12.73	22.01	15.93	0.001*
	*V*_20Gy_	15.62	11.82	18.71	13.83	0.002*

The * presents a p‐value less than 0.05.

**FIGURE 5:**
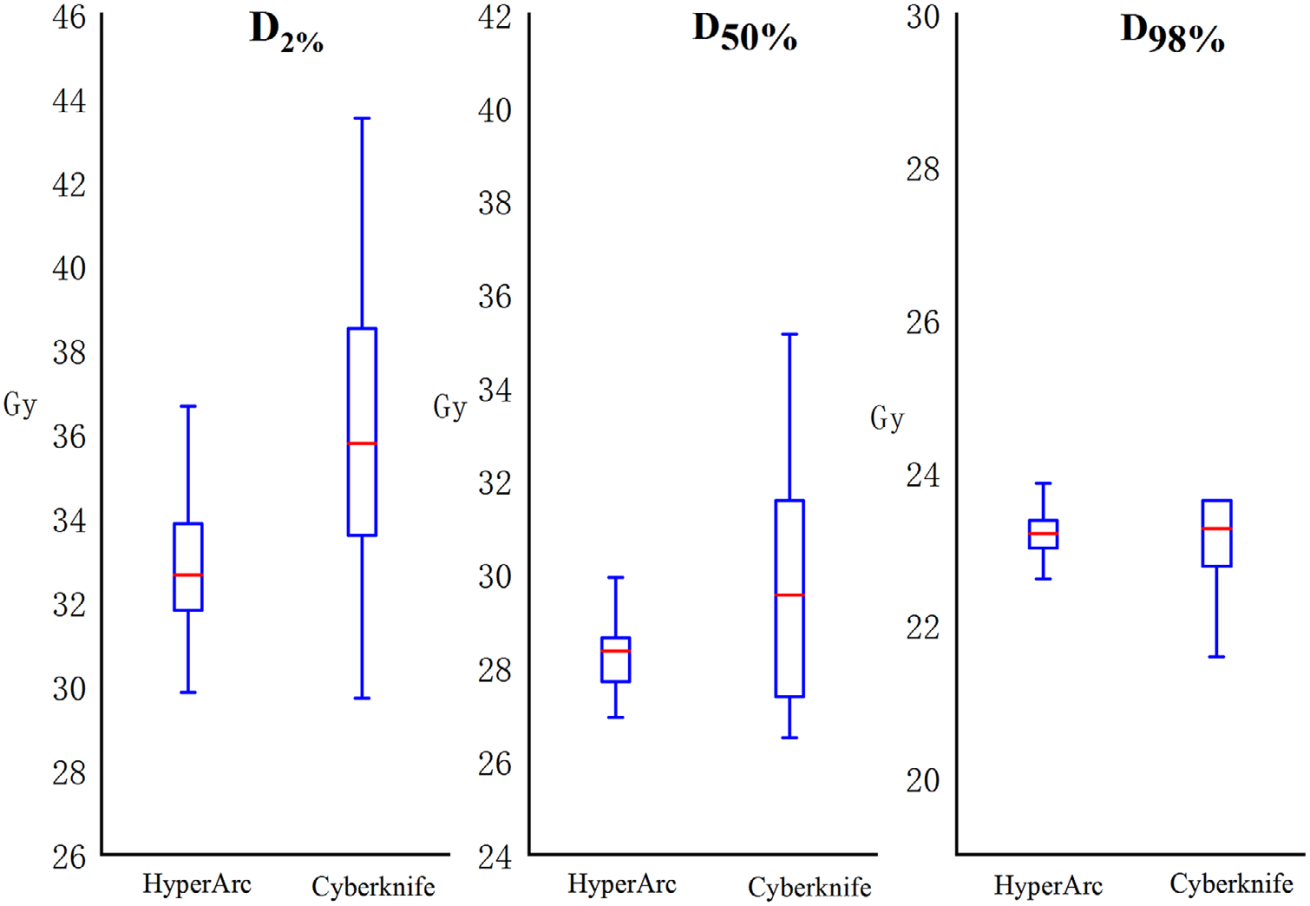
Boxplots of dosimetric parameters of *D*_2%_, *D*_50%_, and *D*_98%_ for tumor volume of HA and CK plans.

Figure 5 indicates that for *D*_2%_ (33.01 ± 1.93 for HA vs. 36.09 ± 3.46 for CK) and *D*_50_% (28.20 ± 0.79 for HA vs. 30.27 ± 3.37 for CK), CK plans were significantly higher than HA plans. However, for *D*_98%_ (23.19 ± 0.41 for HA vs. 23.74 ± 1.97 for CK), no significant difference was noted.

**FIGURE 6:**
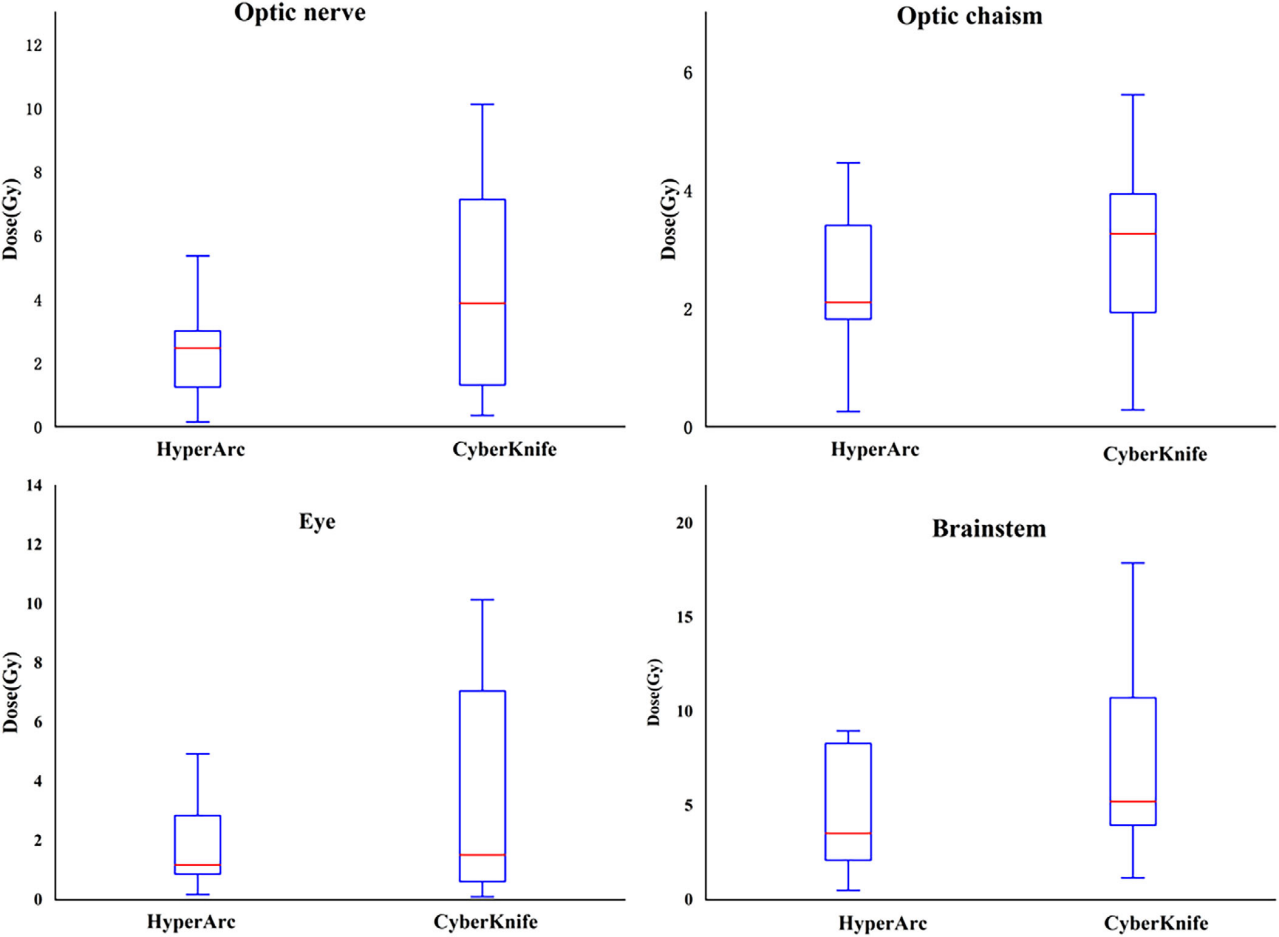
Boxplots of dosimetric parameters for *D*_max_ in optic nerve, optic chiasma, eye and brainstem of HA and CK plans.

Regarding sparing of OARs and normal tissue (NT), Table 2 and Figure 6 show that HA provided better protection than CK for the optic nerve (2.68 ± 1.92 for HA vs. 4.22 ± 3.24 for CK), optic chiasma (2.71 ± 1.82 for HA vs. 3.78 ± 2.96 for CK), and brainstem (6.59 ± 6.75 for HA vs. 8.32 ± 6.94 for CK). No significant difference was noted in sparing the eyes (2.03 ± 1.98 for HA vs. 3.47 ± 3.55 for CK). Additionally, HA plans were more effective in protecting normal brain tissue, with a smaller volume exposed to *V*_2Gy‐20 Gy_ than CK plans.

**FIGURE 7:**
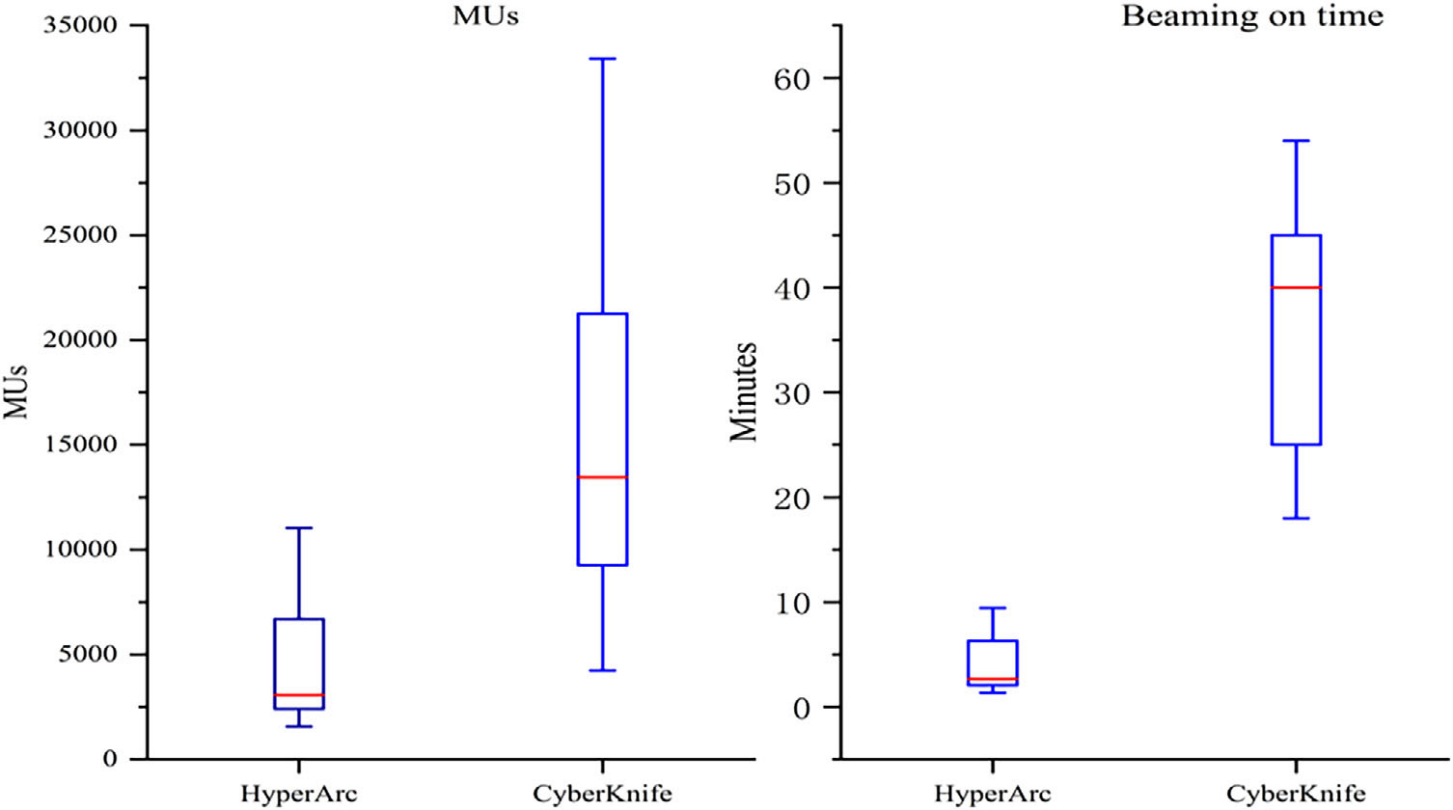
Boxplots of dosimetric parameters for MUs and beaming on time of HA and CK plans.

Furthermore, from Figure 7, the average beaming on time for CK, as estimated by Precision, was 36.41 ± 11.59 min, while for HyperArc plans, estimated by Eclipse, it was 4.03 ± 2.58 min. The average MUs were significantly lower for HA plans (4619.29 ± 2949.67 MUs) compared with CK plans (15 524.60 ± 8607.41 MUs).

**FIGURE 8:**
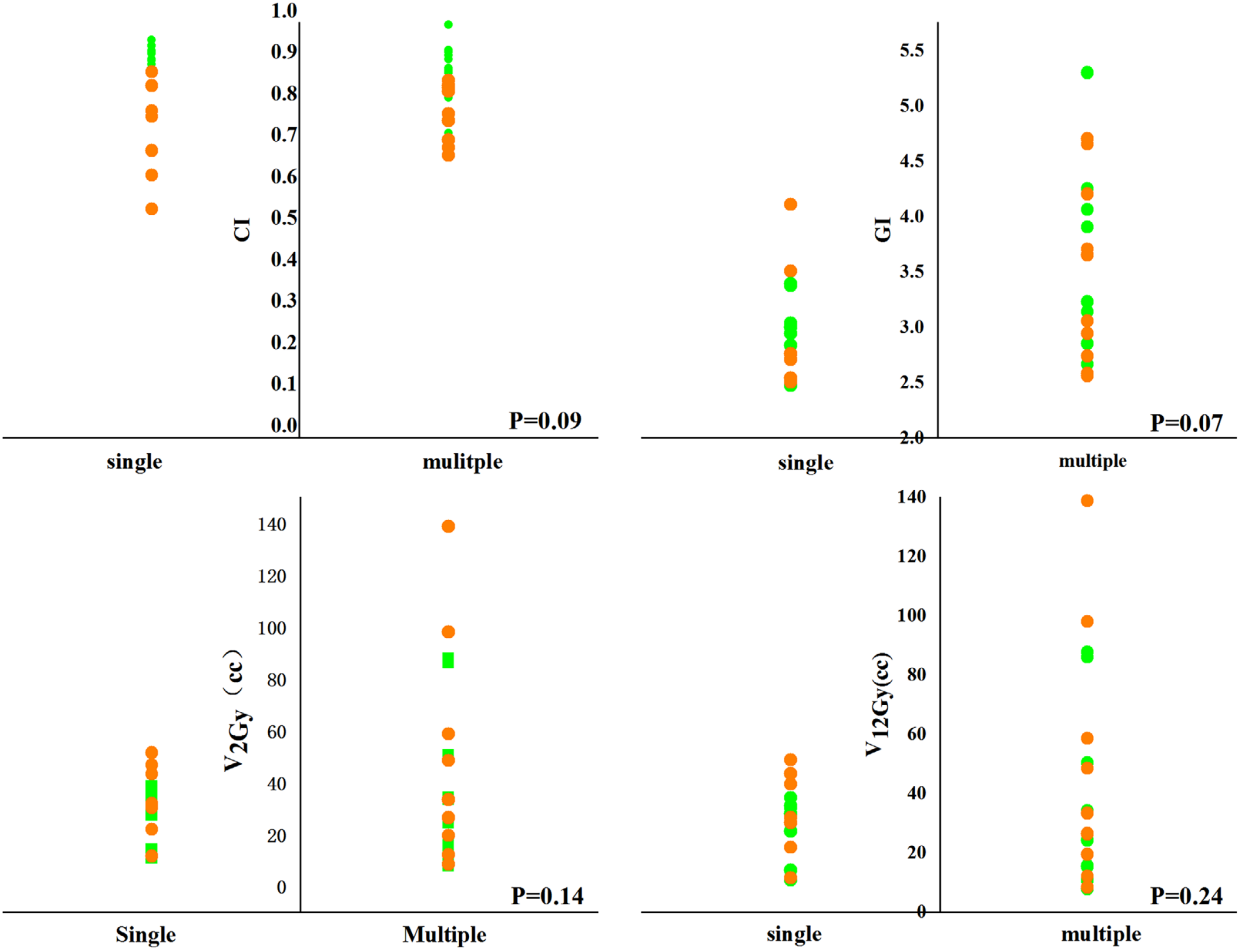
Scatter diagrams of HA and CK plans in CI, GI, *V*_2GY_, *V*_12Gy_ with X axis represents whether is single or multiple metastasis. Green spots represent the HA plans and orange spots represent the CK plans.

A further point biserial analysis between cases with single and multiple lesions was conducted to determine the dosimetric differences within each group. As shown in Figure 8, the coefficients for CI, GI, *V*_2Gy_, and *V*_20Gy_ are 0.43, 0.45, 0.37, and 0.44, respectively.

## 4 DISCUSSION

The treatment of brain metastases using radiotherapy is well‐established.^30^ In SRS, traditional techniques such as GK, CK, or x‐ray Knife, which utilize circular collimators, are believed to better protect normal brain tissue. However, the advent of image‐guided radiation therapy (IGRT) techniques, coupled with advancements in immobilization devices, and notably, the use of thinner multileaf collimators (MLC), have demonstrated the potential of MLC‐based techniques such as helical arc therapy (HA) in treating patients with brain metastases.

Previous comparisons between CK and HA utilized circular collimators. In this study, we compared the performance of HA and CK techniques equipped with MLC in treating brain metastases to mitigate bias. Dosimetric parameters evaluated included HI, CI, GI, *D*_2%_, *D*_50%_, *D*_98%_ of the target, maximum dose (*D*_max_) of OARs, and *V*_2Gy_‐*V*_20Gy_ of normal tissue, and MUs and treatment time. HA plans demonstrated superior CI performance (0.91 vs. 0.77 for CK) and significantly better protection for critical organs, except the eyeballs. Conversely, CK achieved higher radiation doses within the target, evidenced by a higher HI of 1.54 compared to 1.39 for HA and a higher *D*_2%_ with 2 Gy. HA plans also spared more normal tissue from *V*_4Gy_ to *V*_20Gy_, although the GI of CK and HA were similar, exhibiting no significant differences (*p* > 0.05). These results align with those of Kadoya and Ueda, who noted that HA spared more normal tissue (NT) and demonstrated higher GI and CI values. However, our research demonstrates an improvement in the CI of MLC‐based CK plans to 0.77 from 0.60 with circular cones.^22^, ^23^

**FIGURE 9:**
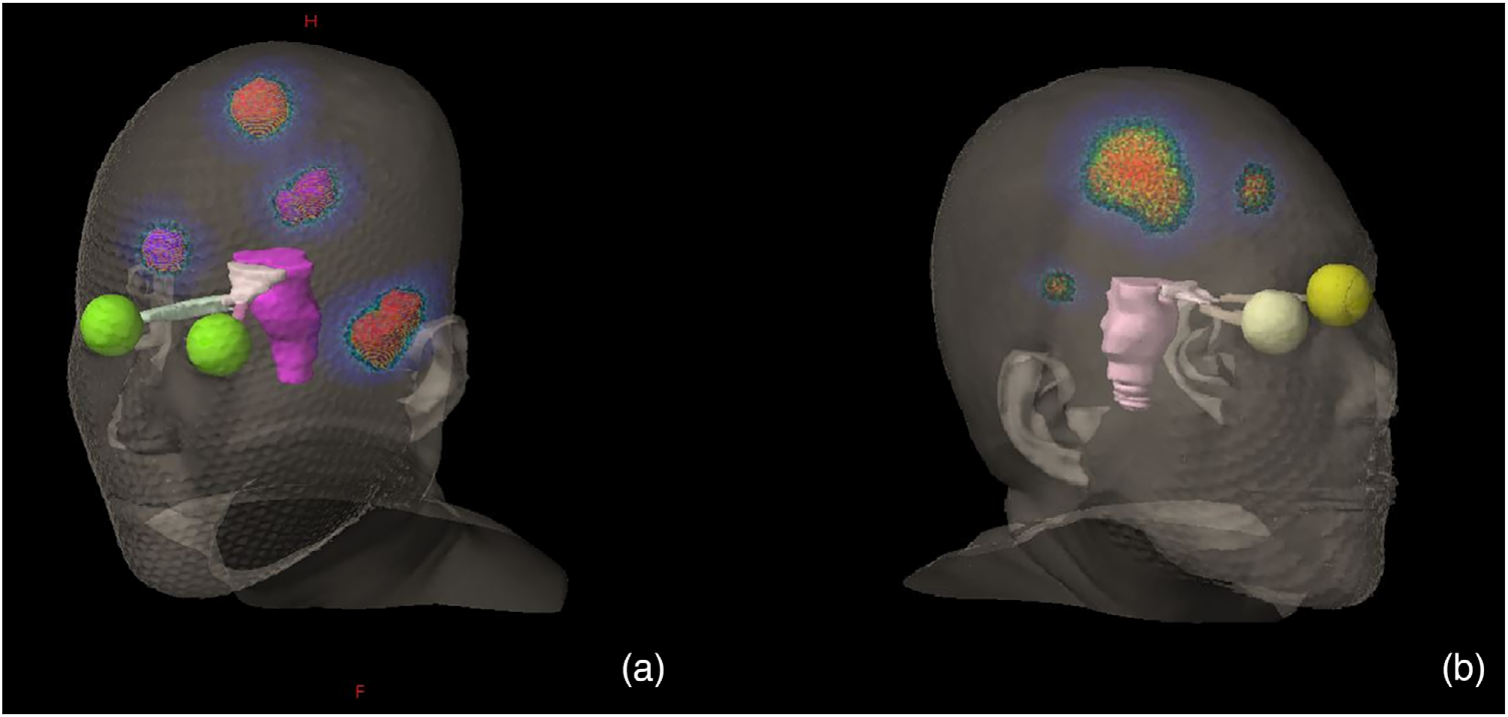
Case No.16 with four scattered and irregular lesions (a) and case No.13 with largest tumor volume (b) and their dose mesh of *V*_12Gy_ are visualized.

HA plans exhibited better performance from *V*_2Gy_ to *V*_20Gy_ in sparing normal tissue, as detailed in Table 1. However, in case No. 16, both techniques performed poorly, although the HA plan significantly exceeded the CK plan with a *V*_12Gy_ of 80 cc compared to 140 cc. As depicted in Figure 9a, unlike case No.13 with largest volume of 35.5 cc in Figure 9b, case No. 16 involved four lesions with irregular shapes and a relatively larger volume of 28.7 cc. scattered from the parietal lobe to the cerebellum, which hindered dose conformity. The CK plans worsened as more angles were required for irradiation, suggesting that the inter‐target distances of lesions might affect dosimetric performance, warranting further studies with more extensive and scattered cases.

Additionally, a point biserial analysis was conducted to discern dosimetric differences between single and multiple lesion cases. Statistical results indicated no significant differences, and Figure 8 classifies the CI, GI, *V*_4Gy_, and *V*_12Gy_ for CK (orange points) and HA (green points), demonstrating random distribution across the groups, a finding consistent with Guinement's research.^24^

Regarding target doses, CK plans achieved higher doses in the planning target volume (PTV), corroborating the findings of Kadoya and Slosarek, where CK plans exhibited higher *D*_max_, *D*_min_, *D*_mean_, and HI in the PTV.^21^, ^22^ This is likely due to the use of only predefined segments in the sequential optimization process, which failed to adequately control the inner dose region, thus delivering higher doses to the target.^31^ Treatment time and MU comparisons were consistent with Ruggieri's findings, indicating that HA plans were more efficient than CK plans, requiring shorter time and fewer MUs.^17^ The total treatment time for HA includes beaming on time (4.03 ± 2.58 min), setup time, and CBCT‐guided verification. Based on our center's experience, the setup is estimated to take 3–5 min and verification 4–5 min.^18^ Although the fixation process for HA is more complex, the overall treatment time for HA plans is significantly shorter than that for CK plans (36.41 ± 11.59 min). Shorter treatment times can enhance patient comfort, reduce positional uncertainty, and increase the cost‐effectiveness of radiation oncology centers.

In SRS, setup error, particularly rotational errors in single iso‐center non‐coplanar plans, is critical.^32^ Ohira's comparison of intra‐fractional motion errors (IME) using open and full‐face masks suggested adding a 1 mm margin to compensate for errors in both groups. However, the American Association of Physicists in Medicine (AAPM) Task Group 135 report recommends a tolerance of less than 0.95 mm for end‐to‐end examinations in monthly quality assurance of robotic radiosurgery systems.^33^ Additionally, CK's real‐time kilovoltage (kV) imaging capabilities allow for automatic correction of IME for more precise delivery, while HA only utilizes CBCT before and after radiotherapy.

This study, while comprehensive, acknowledges limitations that necessitate further investigation. Although a comparison between MLC‐based CK and HA plans in SRS for brain metastases patients was conducted, differences in MLC width, physical properties such as transmission factor and dose leakage gap (DLG), dose calculation grid, and structural contours in different treatment planning systems (TPS) could slightly affect dose‐volume histogram (DVH) statistics.^34^ Furthermore, the current use of a sequential optimizer at our center contrasts with the superior fluence optimization offered by VOLO, which could enhance plan quality, reduce complexity, and shorten treatment time.^35^, ^36^, ^37^ Upgrading the TPS could significantly improve Incise‐based plans. Despite the reliability and safety of both HA and CK techniques, as confirmed by previous dose validation studies and our quality assurance records, a more comprehensive retrospective analysis is anticipated to compare gamma pass rates in dose verification, inter‐fraction and intra‐fraction setup uncertainties, and overall survival (OS), progression‐free survival (PFS), and outcomes such as radionecrosis.^38^, ^39^

## 5 CONCLUSION

In this study, two MLC‐based techniques (CK and HA) in SRS for single and multiple brain metastases had been compared in dosimetric quality. The results showed that both CK and HA can meet the requirements in dose coverage for the target and constraints for OARs, but HA plans can spare NT better and is more suitable for larger lesions, while CK produce a higher dose to targets. And HA owned potential in radiosurgery considering its efficiency and cost‐effectiveness.

## AUTHOR CONTRIBUTIONS

Liying Zhu collected and analyzed the data, and Shengnan Dong drafted the manuscript. Lei Sun designed the study and revised the work. Yixuan Xiao, Yihua Zhong helped the data acquisition and collection. Mingyuan Pan and Yang Wang made the final approval of the version to be published, and are the co‐corresponding authors.

## CONFLICT OF INTEREST STATEMENT

The authors declare no conflicts of interest.

## ETHICS STATEMENT

All the patients have signed the informed consent prior treatment. And this study was approved by our institutional review board (IRB).
